# Library‐Assisted Evolution in Eukaryotic Cells Yield Adenine Base Editors with Enhanced Editing Specificity

**DOI:** 10.1002/advs.202309004

**Published:** 2024-06-14

**Authors:** Shenlin Hsiao, Shuanghong Chen, Yanhong Jiang, Qiudao Wang, Yang Yang, Yongrong Lai, Tao Zhong, Jiaoyang Liao, Yuxuan Wu

**Affiliations:** ^1^ Shanghai Frontiers Science Center of Genome Editing and Cell Therapy Shanghai Key Laboratory of Regulatory Biology Institute of Biomedical Sciences and School of Life Sciences East China Normal University Shanghai 200241 China; ^2^ Department of Hematology The First Affiliated Hospital of Guangxi Medical University Nanning Guangxi 530021 China; ^3^ YolTech Therapeutics Shanghai 201199 China

**Keywords:** adenine base editor, precise gene editing, library‐assisted evolution

## Abstract

The current‐generation adenine base editor (ABE) ABE8e, which has evolved from the prokaryotic evolution system, exhibits high efficiency in mediating A‐to‐G conversion and is presumed to be promising for gene therapy. However, its much wider editing window and substantially higher off‐target editing activity restricted its applications in precise base editing for therapeutic use. This study uses a library‐assisted protein evolution approach using eukaryotic cells to generate ABE variants with improved specificity and reduced off‐target editing while maintaining high activity in human cells. The study generated an expanded set of ABEs with efficient editing activities and chose four evolved variants that offered either similar or modestly higher efficiency within a narrower editing window of protospacer position ≈4–7 compared to that of ABE8e in human cells, which would enable minimized bystander editing. Moreover, these variants resulted in reduced off‐target editing events when delivered as plasmid or mRNA into human cells. Finally, these variants can install both disease‐suppressing mutations and disease‐correcting mutations efficiently with minimal undesired bystander editing making them promising approaches for specific therapeutic edits. In summary, the work establishes a mutant‐library‐assisted protein evolution method in eukaryotic cells and generates alternative ABE variants as efficient tools for precise human genome editing.

## Graphical abstract

ABE variants with high editing efficiency and specificity are obtained through the designed library‐assisted evolution of TadA8e in the eukaryotic cell system. The resulting candidate variants, compared to ABE8e, demonstrate narrower editing window and reduced bystander efficiency as well as lower off‐targeting activity.

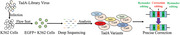

## 1 Introduction

Base editors (BEs), which typically contain a catalytically impaired Cas protein fused to a DNA‐modifying enzyme, can mediate base editing in genomic DNA without DNA double breaks (DSBs), thus safer than nonhomologous ending joining (NHEJ) and homology‐directed repair (HDR) of Cas nuclease‐based method.^[^ ^1^, ^2^ ^]^ The Cas domains were derived from the clustered regularly interspaced palindromic repeats (CRISPR/Cas) systems, such as Cas9 and Cas12, which is in combination with a crRNA‐derived single‐guide RNA (sgRNA) and recognize respective protospacer adjacent motif (PAM).^[^ ^2^, ^3^ ^]^ The different deaminases distinguish various BEs commonly including adenine base editor (ABE) ^[^ ^4^, ^5^, ^6^ ^]^ and cytosine base editor (CBE) ^[^ ^7^, ^8^ ^]^ which enable the transition of A·T to G·C and C·G to T·A, respectively.

The components of common ABE include evolved *Escherichia coli* tRNA adenosine deaminase (TadA) and *Streptococcus pyogenes* Cas9 nickase (spCas9 D10A/nCas9).^[^ ^4^ ^]^ ABE has almost no by‐products (only A to G conversion) in gene editing, which differs from CBE (C to T, G, or A conversions), except for the occurrence of C to G or T base mutations in certain narrow windows (positions 5–7) due to TCN sequence context.^[^ ^1^, ^4^, ^5^, ^6^, ^7^, ^8^, ^9^ ^]^ Moreover, some studies show that CBE has more serious genome‐wide off‐target editing than ABE7.10.^[^ ^10^, ^11^ ^]^ Therefore, ABE is of particular interest for correction of the most common type of pathogenic SNPs in the ClinVar database.^[^ ^1^ ^]^ Notably, ABE8e, which was evolved from ABE7.10 by the phage‐assisted evolution system, has greatly improved deoxyadenosine deamination activity demonstrating much higher efficiency and border editing window compared to ABE7.10. However, ABE8e induces higher off‐target editing activity than ABE7.10. Although V106W mutation ^[^ ^5^ ^]^ and other single amino acid substitutions, like D53E or F148A, could help decrease RNA off‐target,^[^ ^12^ ^]^ all of these mutations could more or less reduce ABE editing efficiency. Thus, continuing efforts in protein engineering are urgently needed to provide TadA variants with minimal off‐target editing efficiency and without compromised on‐target editing activity.^[^ ^5^, ^12^ ^]^ Meanwhile, the ABE8e's wider target window substantially decreases its editing specificity, which reduces precise on‐target single‐base editing for therapeutic edits. The high activity of ABE8e enables correction of the most common type of pathogenic SNPs in the ClinVar database theoretically, such as sickle cell disease (SCD) ^[^ ^13^, ^14^ ^]^ and hereditary hemochromatosis (HH).^[^ ^4^, ^15^ ^]^ However, it is urgently vital to avoid its bystander editing of missense mutation or nonsense mutation before clinical transition.

In this study, considering that ABE7.10,^[^ ^4^ ^]^ ABE8e,^[^ ^5^ ^]^ and ABE8s ^[^ ^6^ ^]^ were evolved by the prokaryotic system, which might result in evolutionary bias not well‐suited for correction of disease‐associated SNPs in eukaryotic genomes,^[^ ^16^, ^17^ ^]^ we performed eukaryotic protein evolution strategy. We screened TadA variants directly using the eukaryotic system and an sgRNA with target adenine embedded in a specific sequence context which may be more beneficial for generating variants with a narrow editing activity window and enhanced specificity in eukaryotic cells. Meanwhile, to overcome the limitation of directed mutation, we used a synthetic screening library which included TadA sequences accessing to deaminase activity domain, making the evolution more frequent and on a much larger scale. We finally generated 4 ABE variants and refer to them hereafter as ABE8e‐Mut14, ABE8e‐Mut15, ABE8e‐Mut25, and ABE8e‐Mut32 (for engineered variants based on ABE8e) which support improved editing specificity and lower byproducts at therapeutically relevant genomic loci in human 293T cells and HepG2 cells. These resulting ABE variants in our study expand the utility of ABEs for precise gene editing.

## 2 Result

### 2.1 Engineering of ABE8e to Narrow Editing Activity Window in Mammalian Cells

**Figure 1:**
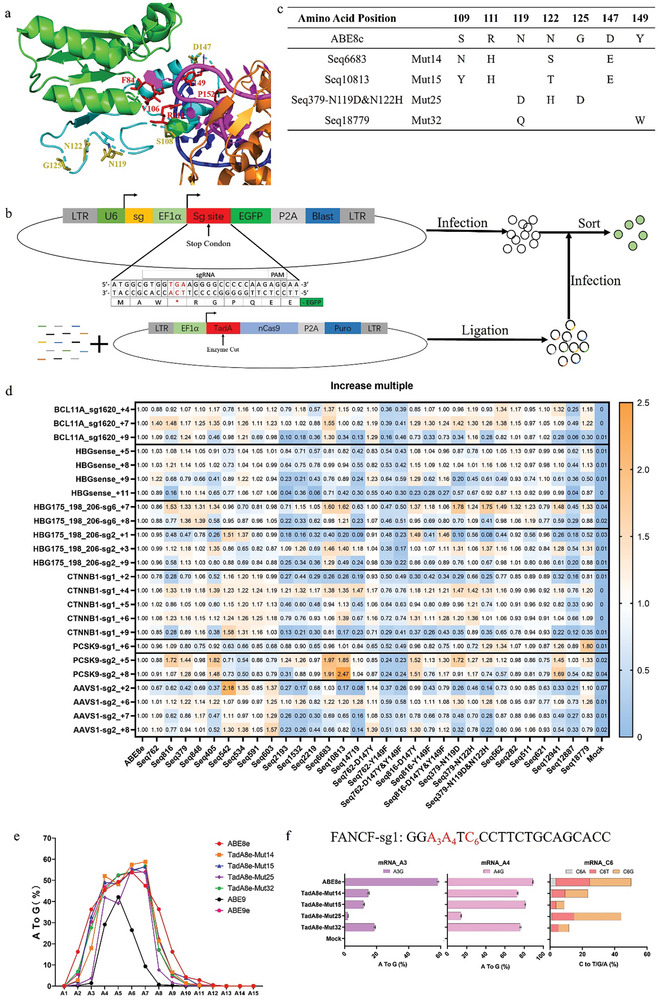
TadA library screening and efficiency plasmid testing. a) The E. Coli TadA8e (green and cyans) structure with spCas9 (orange) and DNA (blue and magenta). The mutant amino acids were red and lemon sticks. The protein data is from RCSB: ABE8e (6VPC). b) The library screening process. LTR is lentivirus parts. EF1α is ABE and EGFP's promotor. U6 is sgRNA's promotor. A BsmBI enzyme cutting site has been designed on TadA. c) The amino acids of Mut14, Mut 15, Mut25, and Mut32 compared to ABE8e. d) ABE8e and 31 mutants’ base editing results in HEK293T at nine genetic sites. Each mutant data is compared with ABE8e to show the A to G efficiency increasing ratio. The base editing frequencies are shown in Figure S4 (Supporting Information). e) Average A‐to‐G editing frequencies of ABE8e, TadA8e‐Mut14, TadA8e‐Mut15, TadA8e‐Mut25, TadA8e‐Mut32, ABE9, and ABE9e. f. Adenine and cytosine editing efficiency of ABE8e, TadA8e‐Mut14, TadA8e‐Mut15, TadA8e‐Mut25, and TadA8e‐Mut32 at FANCF‐sg1 site. All data have three independent biological replicates (mean ± SD).

To alter the editing window of ABE8e without compromising its efficiency, we selected five sites (F84, V106, R111, Y149, P152) ^[^ ^5^, ^18^ ^]^ based on the structure of ABE8e for saturation mutagenesis screening (**Figure** **1**a). Among these sites, R111 and Y149 provided conserved mutations during the previously reported PACE‐assisted bacteria evolution for TadA‐8e and located at the active center. F84, V106, and P152 form a structure conformation critical for the entry of ssDNA into the active site of TadA‐8e. After evaluating genome editing efficiency in HEK293T by plasmid transfection, we found that many mutations resulted in modestly higher efficiency than ABE8e (Figure S1, Supporting Information). We chose two promising mutants, V106K and R111H, for further characterization in human primary cells. Next, we used these two variants to edit human CD34+ hematopoietic stem and progenitor cells (HSPCs) ex vivo through electroporation of either the ABE + sgRNA ribonucleoprotein (RNP) or ABE mRNA with sgRNA. Both of these two variants enable high genome editing without reduction in maximal on‐target activity when electroporated as either mRNA or protein into human CD34+ HSPCs. (Figure S2a–e, Supporting Information). Intriguingly, the ABE8e‐V106K exhibits an editing window shift, with increased efficiency at the +9 position (counting the PAM as positions 21–23) and decreased activity at the +4 position relative to ABE8e (Figure S2a,b, Supporting Information). By investigating the administrating dosage of protein and mRNA, we found that ABE8e‐V106K displayed lower editing efficiency than ABE8e when used at low doses while matched or even exceeded ABE8e's editing efficiency at high doses (Figure S2d,e, Supporting Information). These findings suggested that certain mutations could narrow ABE8e's editing windows without compromising its maximal on‐target activity in mammalian cells. Therefore, to generate new ABE variants that localize deamination activity to a narrow target window within the mammalian genomes, we further accelerate the evolution by developing a eukaryotic screening system that accesses a broader range of TadA sequences.

### 2.2 Library‐Assisted Evolution and Rational Engineering of TadA

We designed a two lentiviral plasmid screening system in the K562 cell line that links the activation of enhanced green fluorescent protein (EGFP) to not only the editing activity but also the editing specificity of a protein from the ABE variant library (Figure 1b). One plasmid of the evolution system designated as P1 expresses ABE variants based on ABE8e vector possessing a synthetic TadA sequences library (Agilent) tethered to SpCas9 nickase (D10A, nCas9). The synthesized TadA sequence library consists of three sub‐libraries, one sub‐library contains single amino acid substitution to each of all 20 canonical amino acids at positions 106–157 of TadA‐8e, another contains site saturation mutagenesis at positions 111, 119, 149, and a third contains substitutions of amino acids carrying same charge for residues at six positions (109S, 111R, 119N, 122N, 147D, 149Y). The other plasmid used in the evolution system designated as P2 encodes sgRNA and a defective EGFP gene with a 27‐bp sgRNA targeting sequence embedded between the start codon ATG and the EGFP coding sequence. And there is a designed stop codon motif (TGA) locating from position 4 to position 7 and an adjacent adenine locating at position 8 in the protospacer, thus, the ABE variant that can mediate efficient +7 A to G conversion enables EGFP production. We used the prepared P2 lentivirus to infect K562 cells and thus created a cell line named hereafter K562‐STOP‐EGFP. Then K562‐STOP‐EGFP cells were infected by the prepared P1 library lentivirus and the top 5% EGFP‐positive cells were sorted for the following deep sequencing analysis to evaluate the editing activities and to identify the corresponding ABE variants (Figure 1b; Figure S3a–d, Supporting Information). Following the biological triplicate library screenings, we identified 22 mutants that ranked top ten. Inspired by the ancestral reconstruction theory,^[^ ^19^ ^]^ based on the top three TadA‐8e variants carrying single point mutations identified by our screening library, we tried to select the two adjacent amino acids around them that were different from TadA‐7.10,^[^ ^5^ ^]^ and did the single and combined back mutation which resulted in nine variants. Therefore, we collectively generated 31 ABE variants for further studies (Figure 1c; Figure S3e, Supporting Information).

### 2.3 Evaluation of the Editing Efficiency of Candidate ABE Variants

To assess the deamination activity of these selected 31 TadA variants fused to nCas9, we transfected plasmids encoding each base editor into HEK293T cells along with a plasmid encoding a sgRNA that targets a site with a cognate PAM for SpCas9 (NGG) and sequenced the target loci after three days (Figure 1c; Figure S3e, Supporting Information). Among seven tested sgRNA targeting sites, some variants demonstrated large improvements in A•T‐to‐G•C conversion efficiency, showing 1.2–2.0‐fold efficiency compared to that of ABE8e (Figure 1d; Figure S4, Supporting Information). Since the K562‐STOP‐EGFP cells have an adenine from the stop codon which is located at position +7 of the protospacer and ABE‐mediated A•T‐to‐G•C conversion of this adenine enables EGFP production, thus our screening system prefers select variants with efficient editing activity at position +7 from the protospacer. And that might have reduced the attention or consideration of adenine base editing from positions far away from the +7 positions, thus only a small number of these variants, such as Seq 542 and Seq 603, localize deamination activity to a large target window within the mammalian genome. We chose the most promising ABE variants including Seq6683 (Mut 14), Seq10813 (Mut 15), Seq379‐N119D&N122H (Mut 25), and Seq18779 (Mut 32) that restricted deamination activity to a narrower editing window and offered higher A·T to G·C conversion frequency compared to ABE8e for subsequent analysis (Figure 1c,d). TadA8e‐Mut14, TadA8e‐Mut15, TadA8e‐Mut25, and TadA8e‐Mut32 exhibited similar editing frequencies at +4‐+7 position, which were compared to ABE8e. The early research showed that NG‐ABE9e could reduce bystander editing.^[^ ^20^ ^]^ The recently reported ABE9 had a narrow window from +4 to +7,^[^ ^21^ ^]^ but it seemed to sacrifice editing efficiency compared to ABE8e and our candidate mutants (Figure 1e; Figure S5, Supporting Information). These mutants decreased the cytosine deamination activity compared to ABE8e, except TadA8e‐Mut25 (Figure 1f). Since mutations of TadA8e at the DNA‐binding site could strongly favor deoxycytidine deamination,^[^ ^22^ ^]^ however, these four mutants had not shown the ability to improve the C‐to‐T (Figure S6, Supporting Information).

### 2.4 On‐Target and Off‐Target Editing Activity Characterization of ABE Variants Delivered as mRNA with sgRNA by LNP

**Figure 2:**
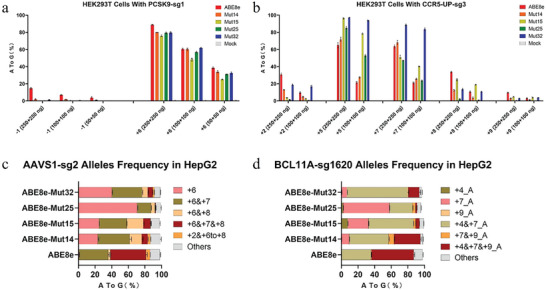
Editing products’ purity analysis of ABE variants when delivered as mRNA by LNP. a,b) Base editing efficiency of different mRNA dosages at a) PCSK9‐sg1 and b) CCR5_UP‐sg3 sites in HEK293T. c,d) Proportion analysis of ABE8e and 4 variants’ editing products at c) AAVS1‐sg2 and d) BCL11A‐sg1620 sites in HepG2. All data have three independent biological replicates (mean ± SD).

Nonviral lipid nanoparticle (LNP) systems which have attractive properties, including reduced immune responses, multi‐dosing capabilities, larger payloads, and flexibility of design, represent the most advanced delivery systems for genetic drugs. Thus we further characterized the editing efficiency and activity windows of Mut 14, Mut 15, Mut 25, and Mut 32 through MC3‐based LNP ^[^ ^23^, ^24^ ^]^ delivery of each ABE mRNA with a sgRNA into HEK293T and HepG2 cells. At all six tested endogenous genome sites, the four candidate ABE variants demonstrated similar or higher editing efficiency than ABE8e at the +5 to +7 position from protospacer while showing reduced editing frequencies at other positions (**Figure** **2**a,b; Figure S7, Supporting Information). Strikingly, amplicon deep sequencing data demonstrated these 4 ABE variants preferred to introduce an A•T to G•C substitution at one single position of the protospacer which is different from ABE8e. In detail, at AAVS1‐sg2 targeting site, we observed these ABE variants offered the largest proportion of single nucleotide editing at +6 position (ABE8e: 0.95%; Mut14: 23.31%; Mut15: 24.95%; Mut25: 70.67%; Mut32: 40.01%), whereas ABE8e provided the largest proportion of dual (34.71%) or triple (47.78%) adenine editing (Figure 2c). Similarly, at the BCL11A‐sg1620 targeting sequence, these variants mainly offered efficient editing activity at +7 single position or +4 and +7 dual positions while ABE8e mainly demonstrated +4, +7, and +9 triple editing (Figure 2d). Notably, the variant ABE‐Mut25 provided a notable narrower editing window with single position editing type accounting for 70.67% at +6 position from AAVS1‐sg2 targeting sequence and 55.47% at +7 position from BCL11A‐sg1620 targeting sequence, respectively (Figure 2c,d). Collectively, these data revealed that the ABE variants yielded from our library‐assisted screening system exhibited improved editing efficiency and narrower editing activity windows.

**Figure 3:**
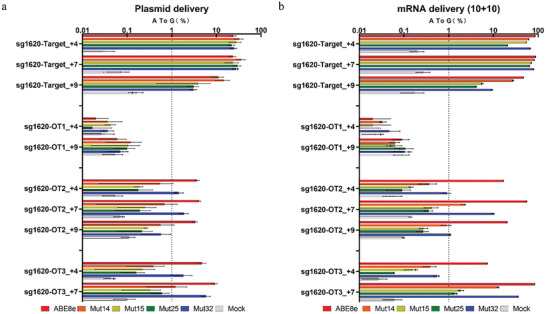
Off‐target editing analysis of ABE variants in the format of plasmid and mRNA. a) Plasmid delivery off‐target data at BCL11A‐sg1620 site in HEK293T cells. b) mRNA delivery off‐target data at BCL11A‐sg1620 in HepG2 cells. All data have three independent biological replicates (mean ± SD).

We used both computational and experimental methods to extensively characterize off‐target (OT) editing as a result of treatment with ABE variant and sgRNA. We have found 3 significant OT sites resulting from ABE8e with sgRNA BCL11A‐sg1620 referring to our previous data.^[^ ^25^ ^]^ Thus, we chose three typical BCL11A‐sg1620 OT sites for evaluation. As anticipated, we did detect evidence of off‐target base editing in edited HEK293T and HepG2 cells compared to negative control cells at three sites. Furthermore, mRNA resulted in more detective off‐target frequencies than plasmids due to much more efficient editing activity of mRNA delivery than plasmid transfection at both on‐target and OT sites (**Figure** **3**a,b; Figure S8, Supporting Information). However, all four variants could reduce the off‐target editing even with a similar or higher on‐target editing efficiency compared to ABE8e when delivered either as plasmids or as mRNA (Figure 3a,b; Figure S8, Supporting Information). In particular, ABE8e‐Mut15 and ABE8e‐Mut25 exhibited significantly lower off‐target editing rates than ABE8e when achieving similar on‐target modification (Figure 3a,b; Figure S8, Supporting Information).

Collectively, we generated four ABE variants, which induce lower off‐target editing and restrict deamination activity to narrower editing windows compared to ABE8e while enabling similar or even modestly enhanced on‐target editing efficiency relative to ABE8eand restrict deamination activity to narrower editing windows compared to ABE8e which might reduce bystander editing and genome‐wide off‐targeting for therapeutic edits.

### 2.5 The Candidate ABE Variants Offer Editing Purity Improvements Over ABE8e for Therapeutic Edits

Finally, we investigated the potential of engineered ABEs to introduce disease‐suppressing mutations and to correct pathogenic mutations in human cells.

Familial hypercholesterolemia (FH) is a frequent hereditary metabolic disease characterized by high serum low‐density lipoprotein (LDL) cholesterol concentration and premature atherosclerotic cardiovascular disease (ASCVD). Since Pcsk9 degrades LDL receptor protein, ABE‐mediated disrupting canonical splice sites of Pcsk9 theoretically enables degradation of Pcsk9 mRNA and protein thus eliminating gene function.^[^ ^26^ ^]^ By lipid nanoparticle‐based delivery of mRNA encoding each ABE variant and a sgRNA targeting the human Pcsk9 splice donor site of intron 1, we observed efficient editing efficiencies, up to ≈80% in 293T cells and even up to ≈100% in HepG2 cells, respectively (Figure 2a; Figure S7a,b, Supporting Information). Intriguingly, engineered ABE variants induced similar on‐target editing +6 position as ABE8e while significantly reducing the unwanted bystander editing at −1 position compared to ABE8e, which indicated the higher precision of engineered ABEs than ABE8e for introducing therapeutic A•T to G•C substitutions (Figure 2a; Figure S7a,b, Supporting Information). Based on these results, we chose those ABE variants for additional characterization about a correction of disease‐associated C•G to T•A substitutions.

**Figure 4:**
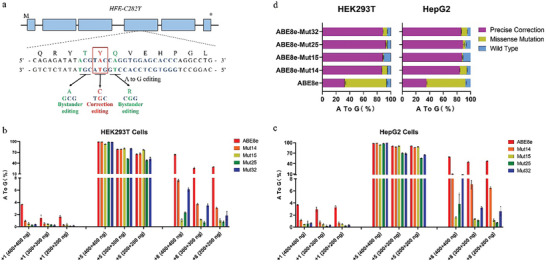
Correction of HFE‐C282Y by ABE variant mRNAs. a) The mutation site of HFE‐C282Y in the human genome and on‐target correction editing as well as possible bystander editing. b,c) Correction outcome in (b). HEK293T and c) HepG2. d) The ratio of precise correction (only +5 position), missense mutation (any type of single or dual or triple editing that contains +1 or +8), and wild type (unedited). All data have three independent biological replicates (mean ± SD).

The iron storage disorder hereditary hemochromatosis (HHC) is an autosomal recessive genetic disorder commonly caused by a G to A mutation at nucleotide 845 in the human HFE gene resulting in a C282Y substitution in the HFE protein that leads to abnormal protein function with excessive iron absorption and potentially life‐threatening elevation of serum ferritin.^[^ ^15^, ^27^ ^]^ There was only one sgRNA with canonical NGG PAM can be used containing the target adenine at the +5 position and two missense mutations at the +1 (ACG→GCG) or +8 (CAG→CGG) position of the protospacer in HHC genome (**Figure** **4**a). However, for this therapeutic edit, ABE7.10 couldn't correct the disease‐associated mutation as efficiently as ABE8e while ABE8e couldn't retain high specificity and enabled substantial A•T to G•C conversion at unexpected +8 position causing missense mutation when induced correction editing at the expected +5 editing position of the protospacer ^[^ ^4^ ^]^ (Figure 4a). To examine the potential utility of engineered adenine base editors for disease correction, we synthesized a short fragment of the HFE gene containing the adenine mutation causing C282Y substitution and integrated it into the genomes of HEK293T and HepG2 cells to create immortalized HHC disease cell models by lentivirus transduction. Then mRNAs encoding ABE variants along with sgRNA that places the target adenine at protospacer position five were delivered into HHC cell models. All the five tested ABE variants demonstrated similar efficient editing activity at the +5 position and nearly undetectable editing activity (<5%) at a position that could lead to missense mutation, but ABE8e enabled especially relatively higher editing efficiency at the +8 position which could also lead to missense mutations (Figure 4b,c). By analyzing the base‐editing product purity, we observed the four candidate ABE variants offered high editing purity (Mut14: 87.12% and 84.11%; Mut15: 88.19% and 87.12%; Mut25: 92.71% and 88.81%; Mut32: 88.60% and 86.17% in HEK293T and HepG2, respectively) while ABE8e induced a large proportion of missense mutations with precise editing lower (32.64% and 35.20% in HEK293T and HepG2 cells, respectively) (Figure 4d). Thus, editing purity of ABE8e was much lower than four candidate ABE variants when single editing frequencies at +5 position were compared to dual editing frequencies at both +5 and +8 positions (ABE8e: 60.92% and 62.37%; Mut14: 92.88% and 90.81%; Mut15: 98.87% and 98.30%; Mut25: 97.68% and 96.18%; Mut32: 94.14% and 91.97% in HEK293T and HepG2, respectively) (Figure S9, Supporting Information). Similarly, the editing purity of ABE8e was still lower than the other 4 ABE variants when single editing frequencies at +5 position were compared to dual editing frequencies at both +5 and +1 positions was still lower than the other 4 ABE variants (ABE8e: 96.44% and 96.38%; Mut14: 99.04% and 98.82%; Mut15: 99.47% and 98.50%; Mut25: 99.72% and 99.44%; Mut32: 99.61% and 99.35% in HEK293T and HepG2, respectively) (Figure S9, Supporting Information). These data suggested that our engineered ABEs should be safer than ABE8e for disease‐associated point mutations causing HHC by avoiding bystander editing resulting in missense mutations. Thus, the engineered ABE variants with narrow deamination activity windows should increase the likelihood of providing a high‐specificity adenine base editing approach for this disease‐associated mutation.

In addition, we installed the HBB^S^ disease locus into the HepG2 genome via lentivirus vector (Figure S10a, Supporting Information). Then we used ABE8e‐NRCH and our candidate ABE variants for the target adenine editing at the +7 position and two bystander adenine editing at the +9 and +12 position, which would cause synonymous mutation.^[^ ^14^ ^]^ ABE8e‐NRCH‐Mut14 and ABE8e‐NRCH‐Mut32 showed 95.83% and 95.62% editing efficiency compared to 98.10% of ABE8e at the +7 position, but bystander efficiencies of ABE8e‐NRCH‐Mut14 and ABE8e‐NRCH‐Mut32 at the +9 position (49.38% and 27.49%, respectively) were significantly lower than ABE8e‐NRCH (93.53%) (Figure S10b, Supporting Information). Moreover, ABE8e‐NRCH‐Mut14 and ABE8e‐NRCH‐Mut32 could reduce the known off‐target editing at eight off‐target sites we further tested ^[^ ^14^ ^]^ (Figure S10c, Supporting Information).

Collectively, we can conclude that these higher‐specificity variants improved base editing product purity and reduced frequencies of undesired editing showing promising therapeutic potential in gene therapy.

## 3 Discussion

The recently evolved TadA‐8e by PACE‐assisted evolution exhibits high deamination activity thus confers ABE8e a wide editing window. Due to the excessively efficient deaminase activity, ABE8e commonly mediates efficient undesired bystander editing and induces substantial off‐targeting efficiency leading to additional pathogenic risks. To circumvent the limitation of efficient ABE8e in specificity and safety, we designed a library‐assisted eukaryotic screening system by modifying the activity domain of TadA and yielded 4 candidate ABE variants with narrower editing activity windows. Moreover, by changing the protospacer sequence, our screening system is also versatile for identifying the most efficient and specific adenine base editors specializing in the corresponding adenine edits in a unique sequence context.

To better understand the enhanced editing specificity of each of the evolved ABE variants at endogenous or even pathogenic sites in human cells, the ABE system was successfully delivered into human cells in the format of mRNA (ABE and sgRNA) utilizing a safer alternative LNP‐delivery method. These activity evaluation experiments demonstrate that the previous state‐of‐the‐art efficient ABE8e showed high activity on both the bystander, off‐target, and cognate motifs, but those engineered ABE variants had drastically reduced bystander alteration and off‐targeting efficiency while they retained near‐ABE8e activity at on‐target editing position. Thus, the development of ABE variants greatly expands the tool chest of high‐specificity adenine base editors and enhances the safety for the installation of therapeutic mutation or correcting pathogenic SNPs that can be addressed by adenine base editing.

Since it was previously reported that mutations reducing the catalytic efficiency of cytidine deaminase in base editors could optimize on‐target precision and reduce bystander and off‐target effects.^[^ ^28^ ^]^ We further reviewed the spatial significance of the substituted residues of TadA‐8e and proposed that the improved specificity of these engineered ABE variants could be owing to substitutions at critical positions for protein‐DNA interaction, such as substitutions at S109, D147, and Y149 that provide steric hindrance and mutations in position R111 that weakens interaction between deaminase and substrate DNA (Figure S11, Supporting Information).

Both library‐assisted screening and PACE are effective methods for protein evolution, while each presents its own set of advantages and disadvantages. Library‐assisted screening can create a large variant library to explore a wide range of diversity in proteins or other molecules, increasing the likelihood of discovering novel functional variants. Additionally, it applies to various biological problems, including but not limited to protein engineering, metabolic engineering, and gene function annotation. However, the quality of screening results highly depends on the quality and diversity of the variant library. Moreover, the large amount of data generated by high‐throughput screening requires complex data analysis tools and skills. On the other hand, PACE can achieve rapid evolution of proteins or RNA molecules in a short period of time. Second, PACE naturally generates diversity through continuous replication and mutation in bacteriophages, eliminating the need to construct complex variant libraries. However, PACE requires special equipment (such as continuous culture systems) and technical expertise, increasing the complexity of experiments. In addition, the success of experiments depends on the ability to accurately set and maintain effective selection pressure, which may limit the optimization of certain properties. Thus, the crucial step is to devise a suitable experimental approach based on the experiment's requirements.

In summary, our rational library‐assisted protein evolution strategy provides new insight for generating gene editors directly from the mammalian screening system, and this approach not only avoids the evolutionary bias of bacteria but tends to yield variants more applicable for gene editing in mammalian cells and at specific sites with unique sequence background.

## 4 Experimental Section

### Molecular Cloning

Fragments encoding ABE variants were amplified using PrimeSTAR Max DNA Polymerase (TAKARA, R045A) or 2×TransStart FastPfu Fly PCR SuperMix (TransGen, AS231) based on the vector backbone of ABE8e (Addgene, #138489).^[^ ^5^ ^]^ The PCR products were purified using the SanPrep Column PCR Product Purification Kit (Sangon Biotech, B518141) and assembled using ClonExpress II One Step Cloning Kit (Vazyme, C112) or ClonExpress MultiS One Step Cloning Kit (Vazyme, C113) according to the manufacturer's protocol. The vector backbone (Addgene, #52963) ^[^ ^29^ ^]^ expressing a guide RNA under the human U6 promoter was digested using FastDigest Esp3I (IIs class) (ThermoFisher, FD0454). The digested products were purified using a Universal DNA Purification Kit (TianGen, DP214). Oligonucleotides for the spacer sequence were ordered from Sangon Biotech and annealed by being heated at 95 °C for 2 min using a PCR instrument. The Esp3I‐digested vector backbone was then assembled with annealed oligonucleotides using T4 DNA Ligase (New England BioLabs, M0202) at room temperature for 30 min. The cloned plasmids were transformed into Trans5α Chemically Competent Cell (TransGen, CD201) for amplification. Then the plasmids were extracted using EndoFree Maxi Plasmid Kit (TianGen, DP117) or EndoFree Mini Plasmid Kit II (TianGen, DP118).

### Cell Culture

HEK293T and HepG2 cells were cultured in Dulbecco's Modified Eagle Medium (DMEM) (Gibco, C11995500BT) with 10% fetal bovine serum (FBS) (Gibco, 10099141 or KEL Biotech, KC001) and 1% penicillin/streptomycin (BasalMedia, S110JV). K562 cells were cultured in Iscove's Modified Dulbecco's Medium (IMEM) (Gibco, C12440500BT) with 10% fetal bovine serum (FBS) (Gibco, 10099141 or KEL Biotech, KC001) and 1% penicillin/streptomycin (BasalMedia, S110JV). Human CD34 + cells were cultured in X‐VIVO 15 (04‐418Q, Lonza) supplemented with 100 ng mL^−1^ recombinant human thrombopoietin (PeproTech, AF‐300‐18), 100 ng mL^−1^ recombinant human stem cell factor (hSCF) (PeproTech, AF‐300‐07) and 100 ng mL^−1^ recombinant human Flt3‐ligand (PeproTech, AF‐300‐19). All cells were cultured at 37 °C with 5% CO_2_.

### Plasmid Transfection

The 50 000–100 000 HEK293T cells were seeded in each well of the 48‐well plates (Corning) and transfected at 60–70% conjunction. The 24 h following seeding, 250 ng base editor plasmid and 250 ng sgRNA plasmid were co‐transfected into HEK293T cells using polyethyleneimine (PEI, Polysciences) according to the manufacturer's protocol. 24 h after transfection, 2 ug mL^−1^ puromycin was added into the media for the following 48 h. Genome DNA was extracted using TIANamp Genomic DNA Kit (TianGen, DP304) 72 h following transfection and amplified by PCR using KOD ‐Plus‐ Neo (TOYOBO, KOD‐401)for sequencing.

### mRNA Transcription In Vitro

The template DNA encoding ABE variants for mRNA transcription was obtained by PCR using KOD ‐Plus‐ Neo (TOYOBO, KOD‐401). The transcription was completed using the mMESSAGE mMACHINE T7 Ultra Kit (Life, AM1345) following the manufacturerʼs instructions. The transcription products were purified by RNeasy Mini Kit (QIAGEN, 74104).

### Protein Purification

Plasmids for protein expression of ABE variants were cloned into the pET28a vector backbone. Proteins of ABE variants were expressed in E. Coli BL21 (DE3) (ThermoFisher), which were grown in LB media at 37 °C and 4–5 h later induced by 1 mm isopropyl ß‐d‐1‐thiogalactopyranoside for 18–20 h at 16 °C. Cells were collected and lysed by sonication in 500 mm NaCl, 20 mm Tris‐HCl (pH 8.0), 1 mm TCEP, and 10% glycerol buffer. The lysate was centrifuged at 10 000 g for 45 min. The supernatant was filtered by a 0.22 um Millex‐GP Syringe filter unit (Millipore) and loaded onto HisTrap HP (GE). The proteins were eluted with a gradient of lysis buffer with 300 mm imidazole. The proteins were further dialyzed with SnakeSkin Dialysis Tubing (10K MWCO, ThermoFisher) for 24 h in the dialysis buffer containing 500 mm NaCl, 20 mm HEPES (7.5), 1 mm TCEP, and 10% glycerol, enabling buffer exchange and low‐molecular‐weight contaminant removal from sample solutions without significant loss of the macromolecule of interest. The dialyzed proteins were concentrated to ≈15 mg mL by Amicon Ultra‐4 Centrifugal Filter Unit (Millipore) and stored at −80 °C.

### RNP and mRNA Electroporation

Electroporation was performed using Lonza 4D Nucleofector (V4XP‐3032 for 20 µL Nucleocuvette Strips) following the manufacturer's instructions. The chemical‐modified synthetic sgRNA (2′‐O‐methyl 3′ phosphorothioate modifications in the first and last three nucleotides) was ordered from GenScript. CD34+ HSPCs were thawed and maintained in an X‐VIVO medium supplemented with cytokines 24 h before electroporation. For 20‐µL Nucleocuvette Strips, the ribonucleoprotein (RNP) complex consisting of ABE protein and chemically synthesized sgRNA was prepared by mixing protein (100 pmol) and sgRNA (300 pmol) and incubating for 15 min at room temperature immediately before electroporation. Fifty thousand HSPCs resuspended in 20 µL of P3 solution were mixed with RNP and transferred to a cuvette for electroporation with program EO‐100. For mRNA 20‐µL Nucleocuvette Strips electroporation, the mRNA complex was prepared by mixing ABE8e mRNA (1 µg) and sgRNA (300 pmol).

### Library Designation

The TadA Library consisting of 23393 variant sequences contained modifications across from position 106 to position 157 and synthesized by Agilent (SurePrint Oligonucleotide Libraries, G7238A). For P1 plasmid construction, the DNA oligonucleotides library of TadA was cloned into Esp3I‐digested Lenti‐ABE8e vector derived from ABE8e vector backbone (Addgene, #138489) and lentivirus vector backbone (Addgene, #52962) ^[^ ^29^ ^]^ using T4 DNA ligase (NEB, M0202L). For P2 plasmid construction, the sgRNA expression fragment and the impaired EGFP gene containing screening‐sgRNA targeting sequence were cloned into vector backbone (Addgene, #52963) and the original puromycin gene was replaced by the impaired EGFP gene.

### Creating a Stable Cell Line of K562‐STOP‐EGFP or Disease Model

For the lentivirus package, HEK293T cells were transfected at 70% confluence in 12 mL of medium with 13.3 µg of psPAX2 (Addgene plasmid no. 12260), 6.7 µg of VSV‐G (Addgene plasmid no. 14888) and 20 µg of the lentiviral construct plasmid of interest using 180 µg of PEI. The medium was changed 6 h after transfection. Lentiviral supernatant was collected at 48 h following transfection. The prepared lentivirus was used to infect K562 or 293T cells creating an interest cell line.

### Deep Sequencing Analysis

The top 5% EGFP‐positive K562 cells were sorted by FACSAria (BD BIOSCIENCES) 48 h following the TadA library lentivirus infection. The cell genome was extracted and the interesting fragment was amplified by PCR with a pair of site‐specific primers using KOD ‐Plus‐ Neo (TOYOBO, KOD‐401). Amplicons were sequenced for 2 × 150 paired‐end reads with the MiSeq Sequencing System (Illumina). Frequencies of editing outcomes and related TadA variants were quantified using CRISPResso2 software.^[^ ^30^ ^]^

### LNP Delivery in Vitro

LNP components including Dlin‐MC3‐DMA (Chemicals, DC10800), 1,2‐distearoyl‐sn‐glycero‐3‐phosphocholine (DSPC) (Sigma, 850365P), Cholesterol (Sigma, 700000P) and 1,2‐Dimyristoyl‐rac‐glycero‐3‐methoxypolyethylene glycol‐2000 (DMG‐PEG 2000) (Sigma, 880151P) were dissolved in ethanol. Dissolved components were mixed at a molar ratio of 50: 10: 38.5: 1.5 (MC3: DSPC: Cholesterol: DMG‐PEG 2000) and stored at −20 °C until use. The mRNA and sgRNA of testing dosage (mass ratio 1:1) were mixed in 10 mm citrate buffer (pH 4.0) (total 30 uL). The LNP mixture was added and diluted to the ethanol (total 10 uL). The mRNA/sgRNA mixture was gently added according to the ratio that the N (MC3): P (RNA) ratio was 8:1. The final mixture was incubated for 10 min at room temperature and then diluted in 100 uL DMEM and dropped slowly into 293T or HepG2 cells.^[^ ^23^, ^24^, ^31^, ^32^, ^33^ ^]^

## Conflict of Interest

The authors declare no conflict of interest.

## Author Contributions

S.H., S.C. and Y.J. contributed equally to this work. S.H. planned and performed experiments. S.C. help with design the library. S.C. and Y.J. helped with experiment conductions. Q.W. helped with the generation of synthesized TadA library sequences and analysis of the library deep sequencing data. Y.Y. and Y.L. provided the cell sample collection and the guidance of CD34+ cell culture. T.Z. provided help for this project. Y.W. initiated the project. Y.W. and J.L. supervised the research. S.H., S.C., J.L., and Y.W. wrote the manuscript with input from all the authors.

## Supporting information

Supporting Information

## Data Availability

The data that support the findings of this study are available in the supplementary material of this article.
