# Supplementary material for: Library‐Assisted Evolution in Eukaryotic Cells Yield Adenine Base Editors with Enhanced Editing Specificity
Source: Adv Sci (Weinh). 2024 Jun 14;11(30):2309004. doi: 10.1002/advs.202309004 (PMC11321652; doi:10.1002/advs.202309004)
Supplement: Supplementary file 1 — Supporting Information [file ADVS-11-2309004-s001.docx]

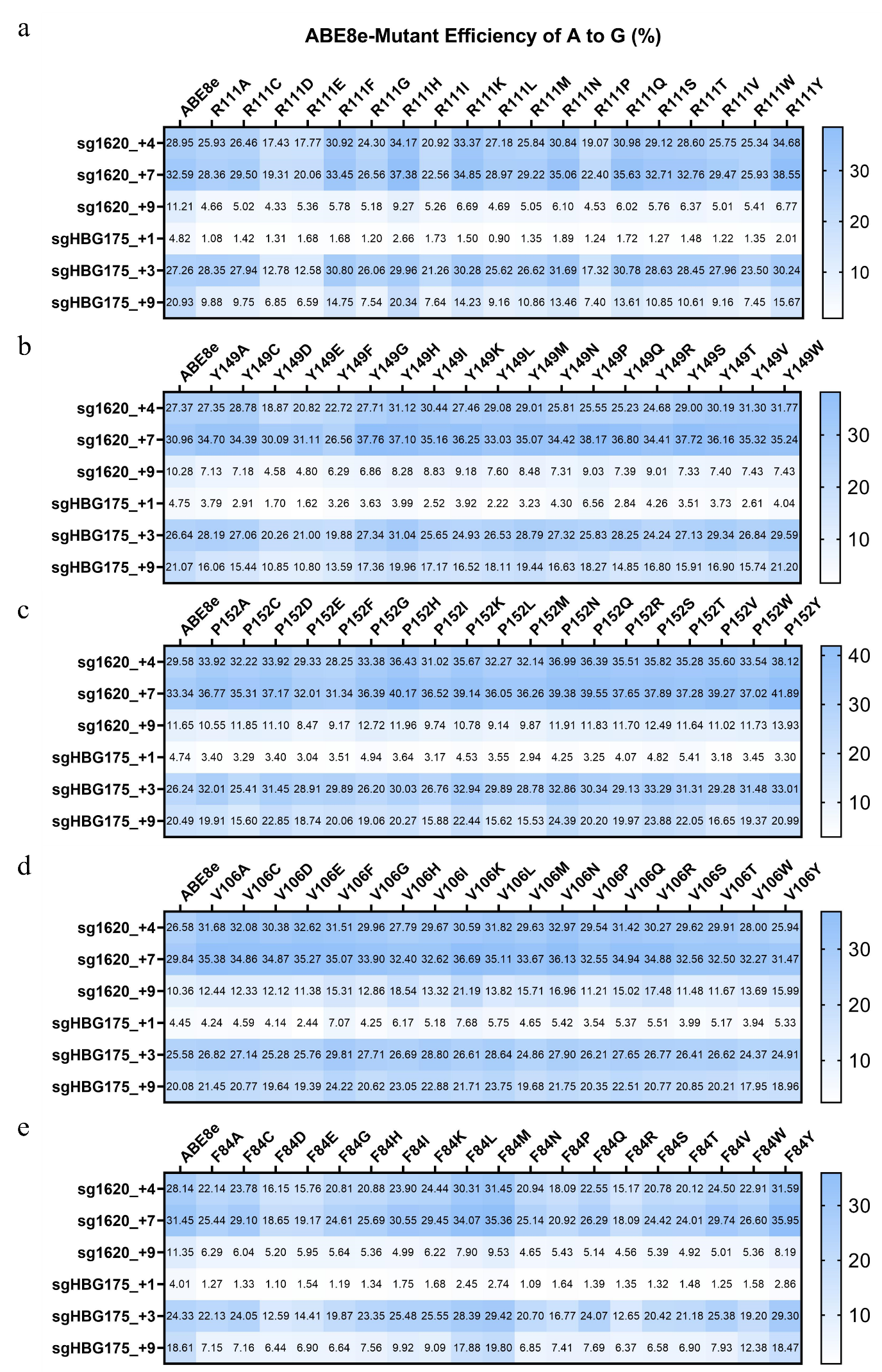


Figure S1 | **TadA engineering by saturation mutagenesis**. a. ABE8e-R111, b. ABE8e-Y149, c. ABE8e-P152, d. ABE8e-V106 and e. ABE8e-F84 mutations’ editing efficiency by plasmid delivery into HEK293T at BCL11A-sg1620 and sgHBG _175 sites. All the data have three independent biological replicates (mean ± SD).


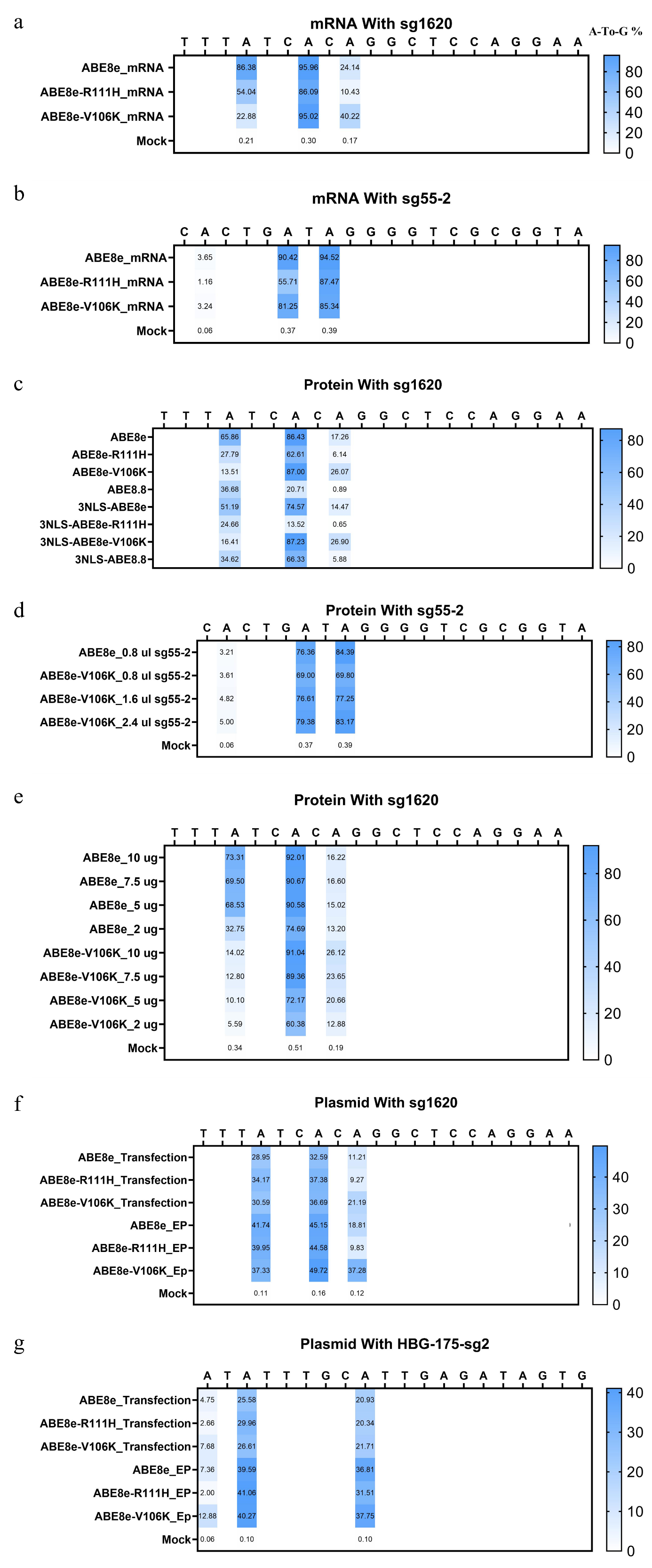


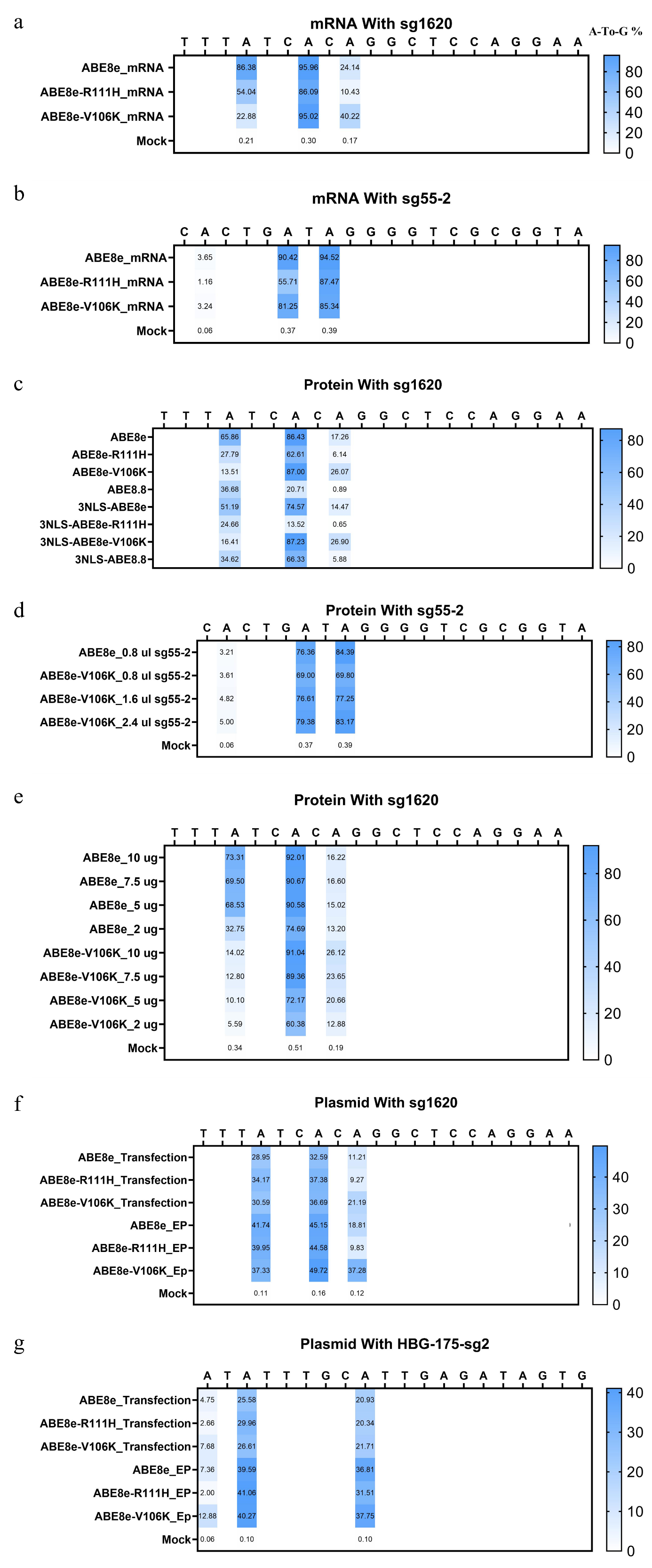


Figure S2 | **ABE8e variants’ editing efficiency by different delivery method.** a-b. Editing efficiency of ABE8e, ABE8e-R111H and ABE8e-V106K at a. BCL11A-sg1620 and b. 55-sg2 site by electroporation as mRNA into CD34+ HSPCs. c. Editing efficiency of (3NLS-)ABE8e, ABE8e-R111H, ABE8e-V106K, and ABE8.8 at BCL11A-sg1620 site when electroporated as protein with sgRNA into CD34+ HSPCs. d-e. Evaluation of ABE8e and ABE8e-V106K protein and sgRNA concentration at BCL11A-sg1620 site in CD34+ HSPCs. f-g. Analysis of editing outcomes of ABE8e, ABE8e-R111H, and ABE8e-V106K by plasmid transfection with PEI and plasmid electroporation (EP) at f. BCL11A-sg1620 and g. sgHBG175_2 site in HEK293T cells.


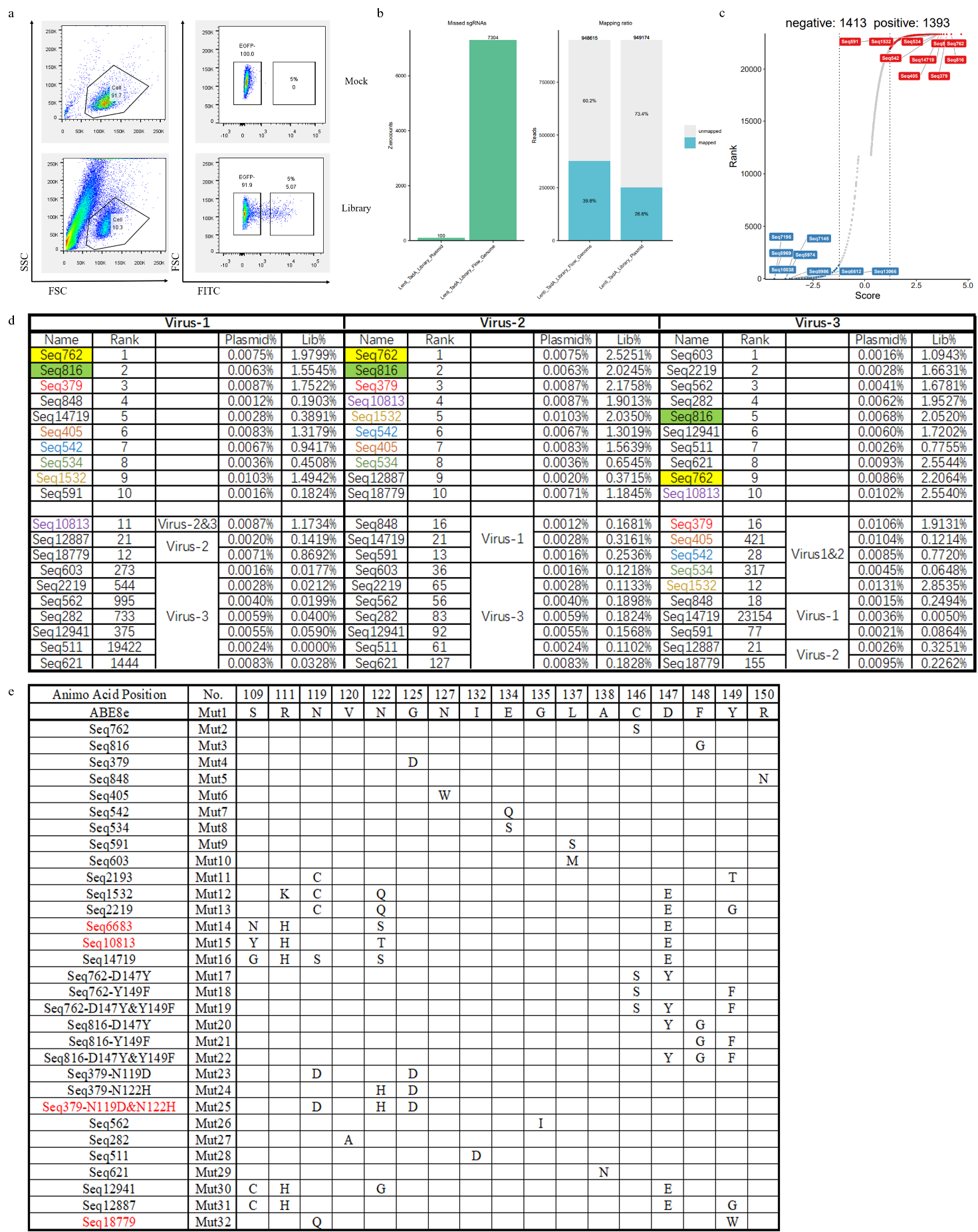


Figure S3 | **Data analysis of TadA library screening.** a. Flow cytometer sorted the top 5% positive EGFP cells of TadA library. b-c. The next-generation sequencing data (one representative of three biological triplicate experiments) analysis showed the rank of ABE mutations. d. The top ten of the ranking list of ABE mutations in the library. The data showed results of three independent biological replicates. e. The list included the top ten variants and their containing mutations. Mut17-Mut25 were restored to original TadA7.10 from TadA8e, at sites that were near Mut1-3’s mutation sites.


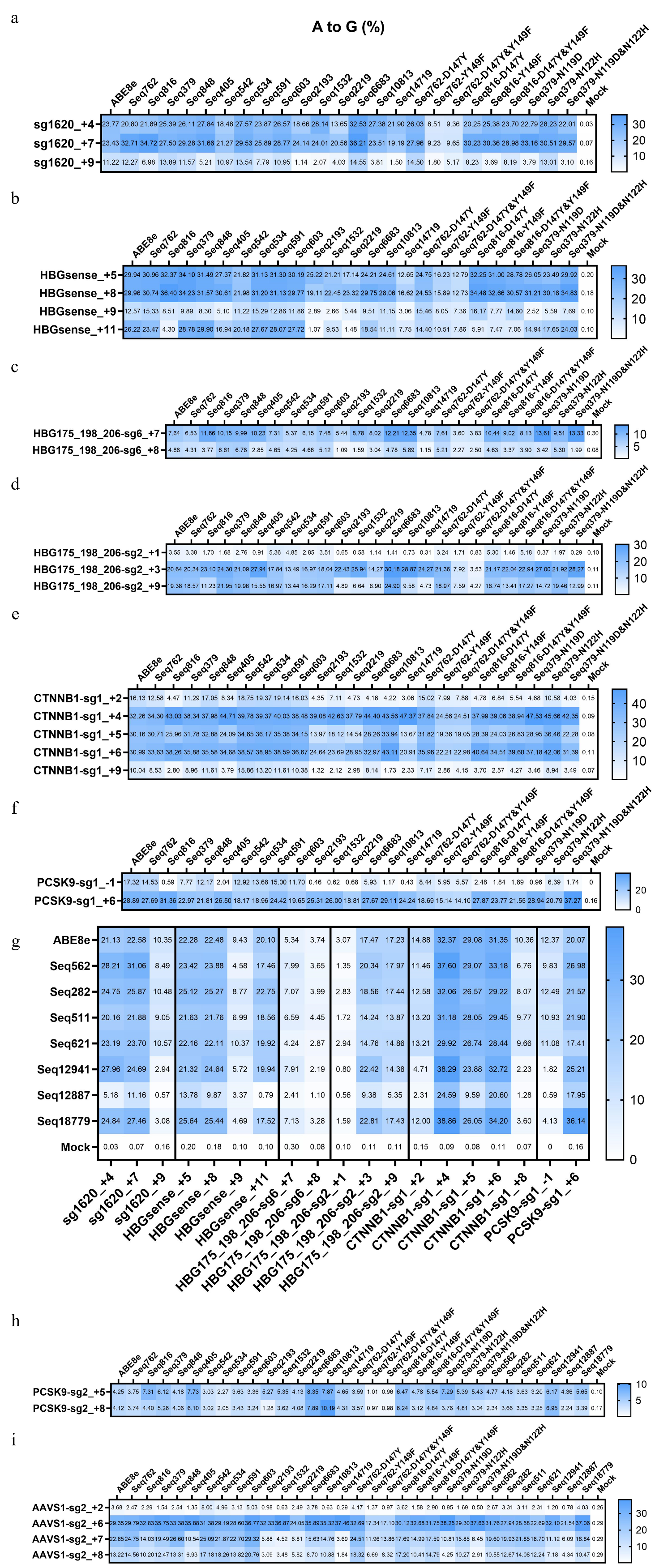


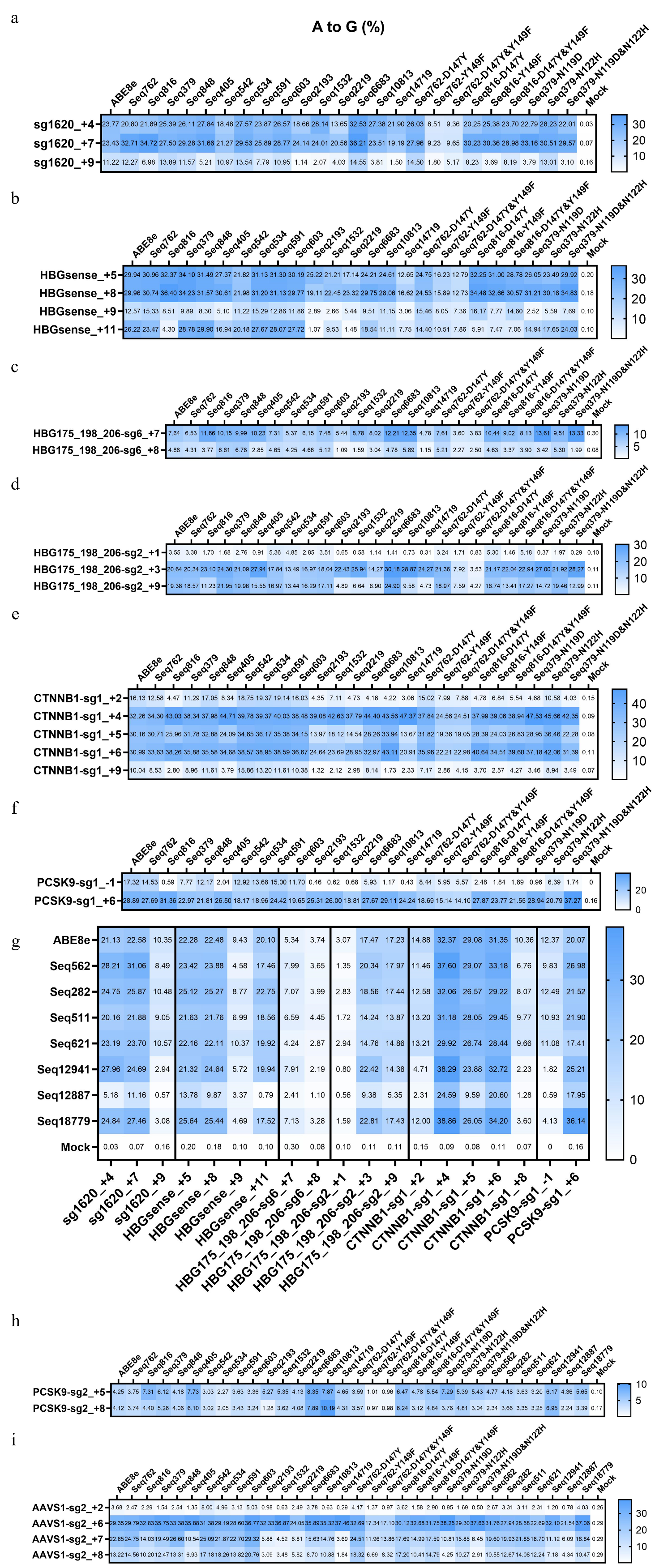


Figure S4 | **Profile of ABE variants’ editing efficiency at endogenous sites with plasmid transfection into HEK293T cells.** a-f. 24 variants’ editing frequency at a. BCL11A-sg1620, b. sgHBGsense, c, HBG175_198_206-sg6, d. HBG175_198_206-sg2, e. CTNNB1-sg1, and f. PCSK9-sg1 sites. g. Mut26 – Mut32 editing frequencies at BCL11A-sg1620, sgHBGsense, HBG175_198_206-sg6, HBG175_198_206-sg2, CTNNB1-sg1, and PCSK-sg1 sites. h-i. 31 variants’ editing frequency at h. PCSK9-sg2 and i. AAVS1-sg2 sites. All the data have three independent biological replicates (mean ± SD).


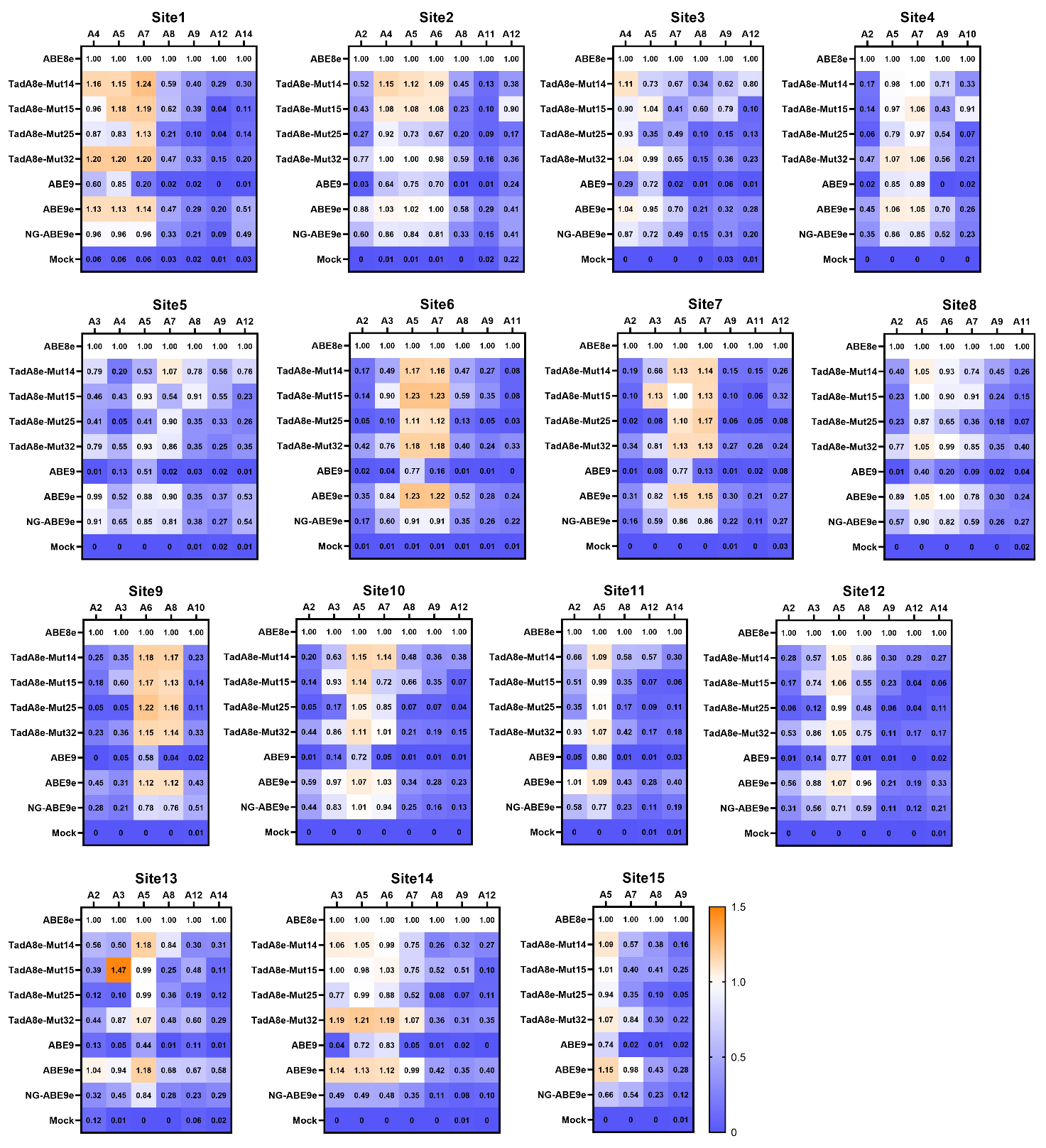


Figure S5 | **Profile of ABE8e, ABE8e variants, ABE9, ABE9e, and NG-ABE9e’s editing efficiency at endogenous sites with plasmid transfection into HEK293T cells.** Each mutant’s editing data is compared with ABE8e to show the A to G efficiency increasing ratio in HEK293T at fifteen genetic sites. All the data have three independent biological replicates (mean ± SD).


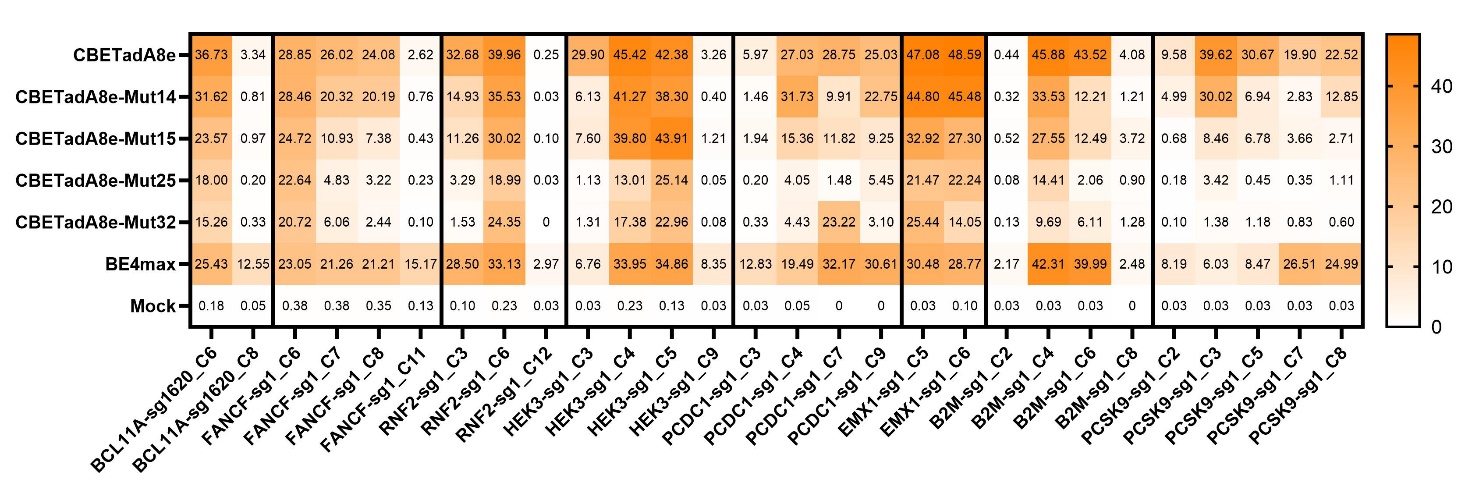


Figure S6 | **Profile of CBETadA8e variants’ editing efficiency at endogenous sites with plasmid transfection into HEK293T cells.** CBETadA8e, CBETadA8e-Mut14, CBETadA8e-Mut15, CBETadA8e-Mut25, CBETadA8e-Mut32 and BE4max editing C-to-T frequency at BCL11A-sg1620, FANCF-sg1, RNF2-sg1, HEK3-sg1, PCDC1-sg1, EMX1-sg1, B2M-sg1, and PCS9-sg1 sites. All the data have three independent biological replicates (mean ± SD).


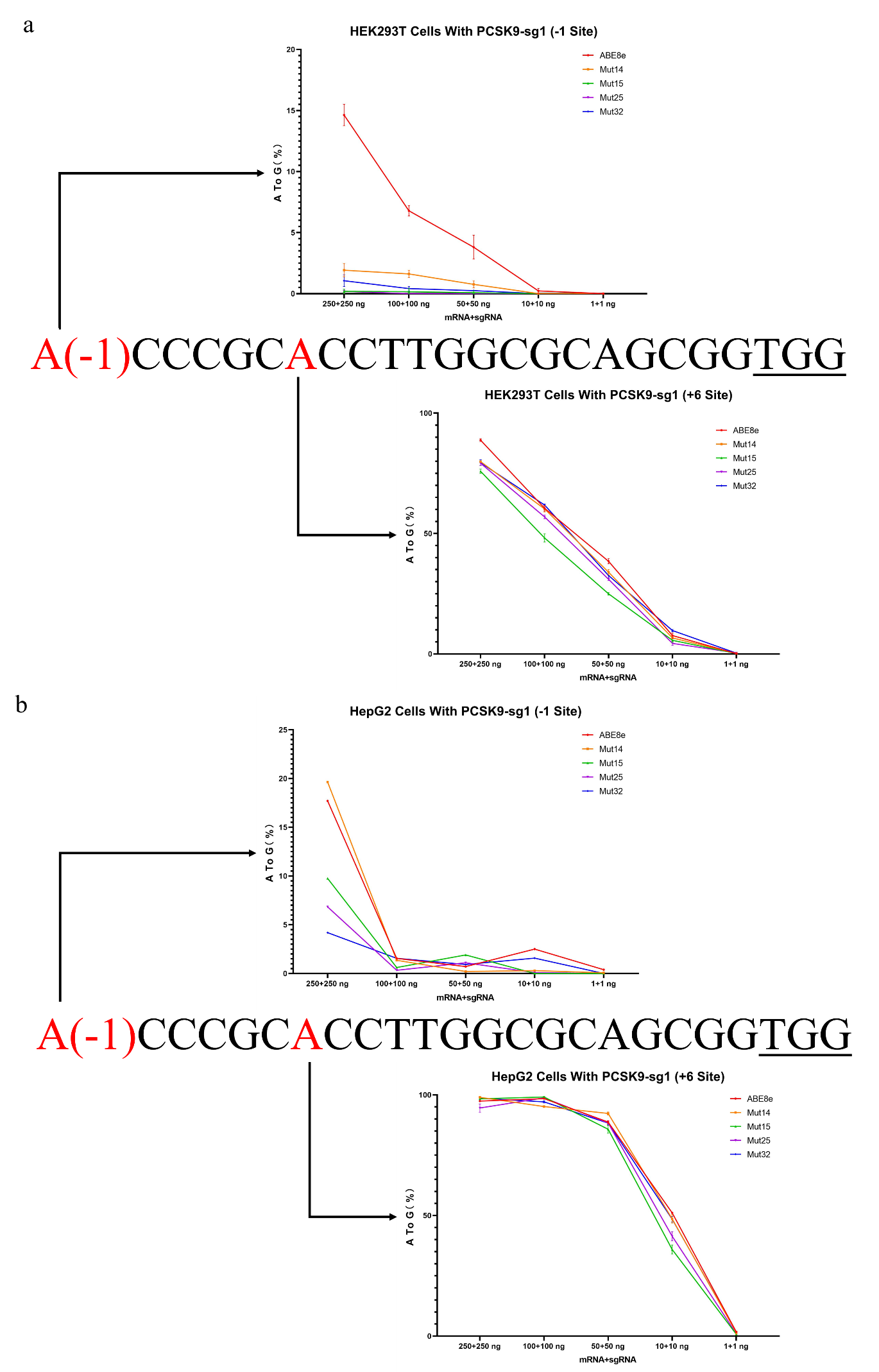


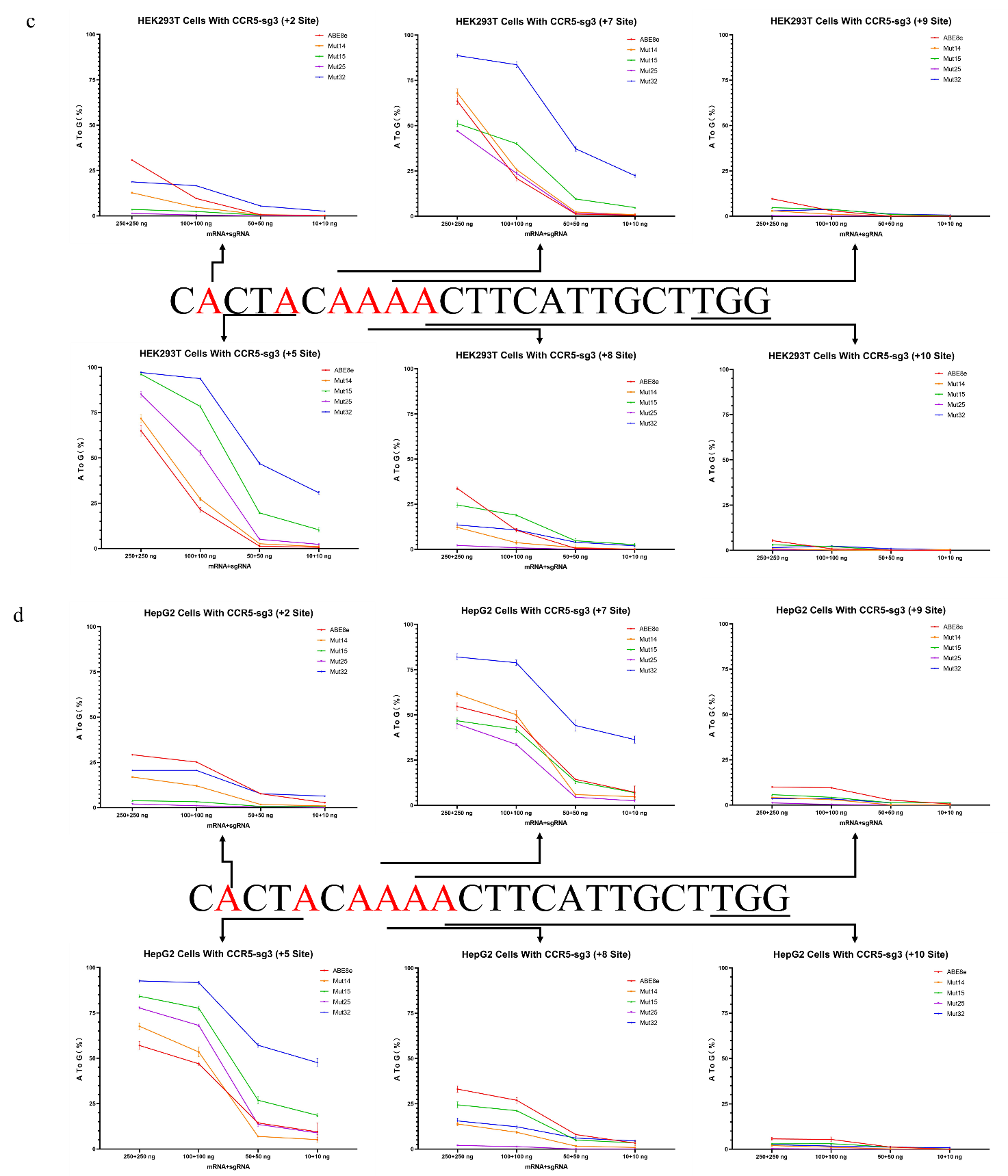


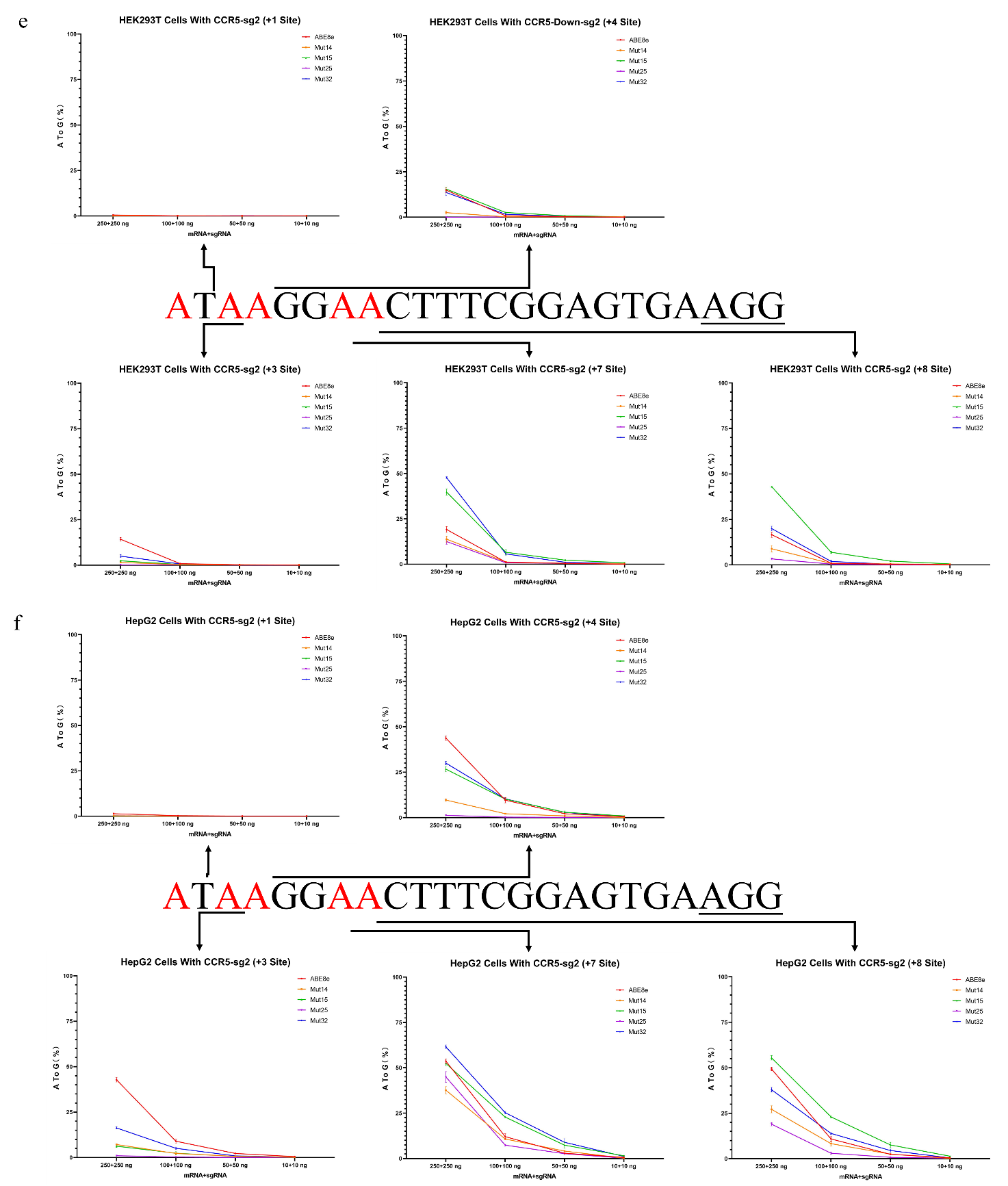


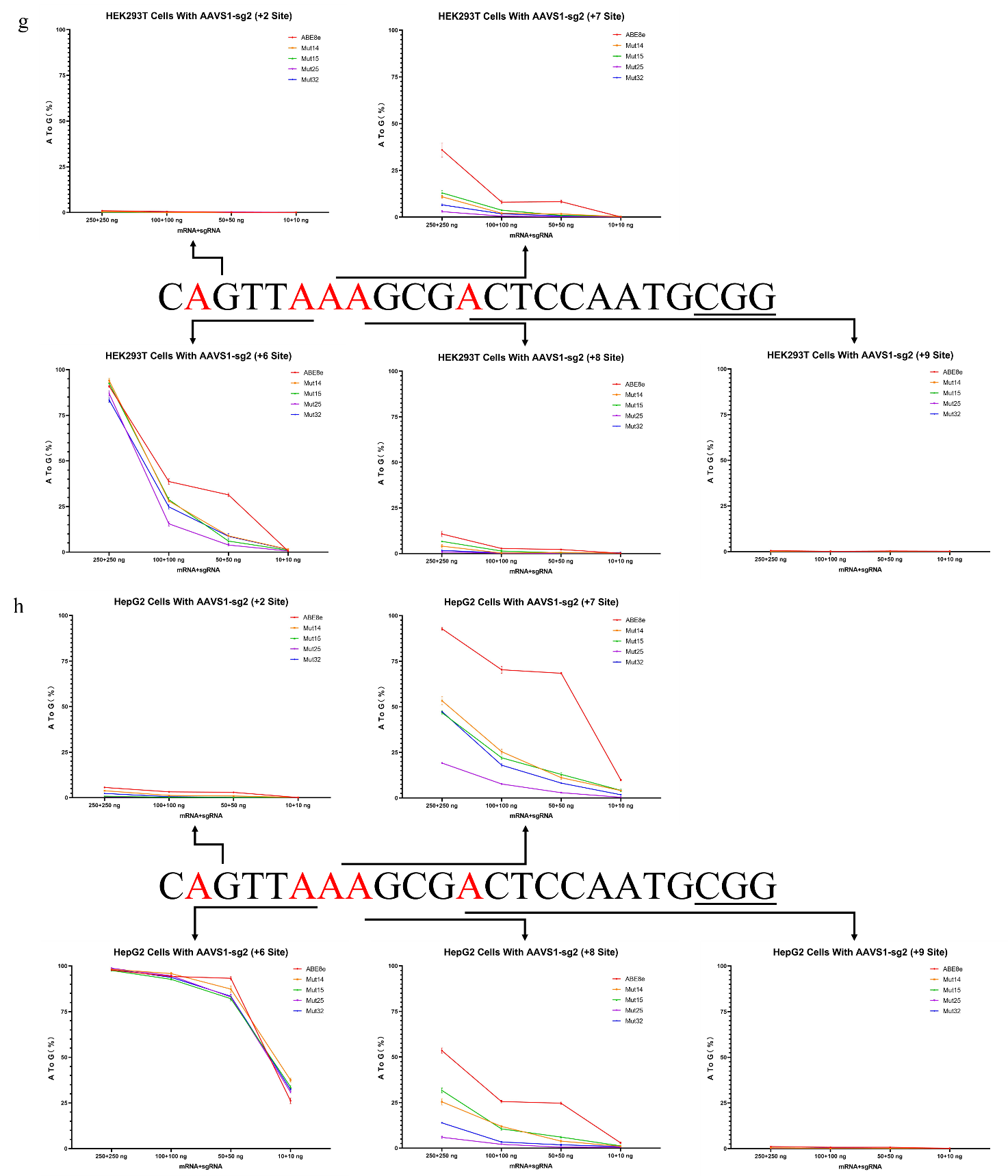


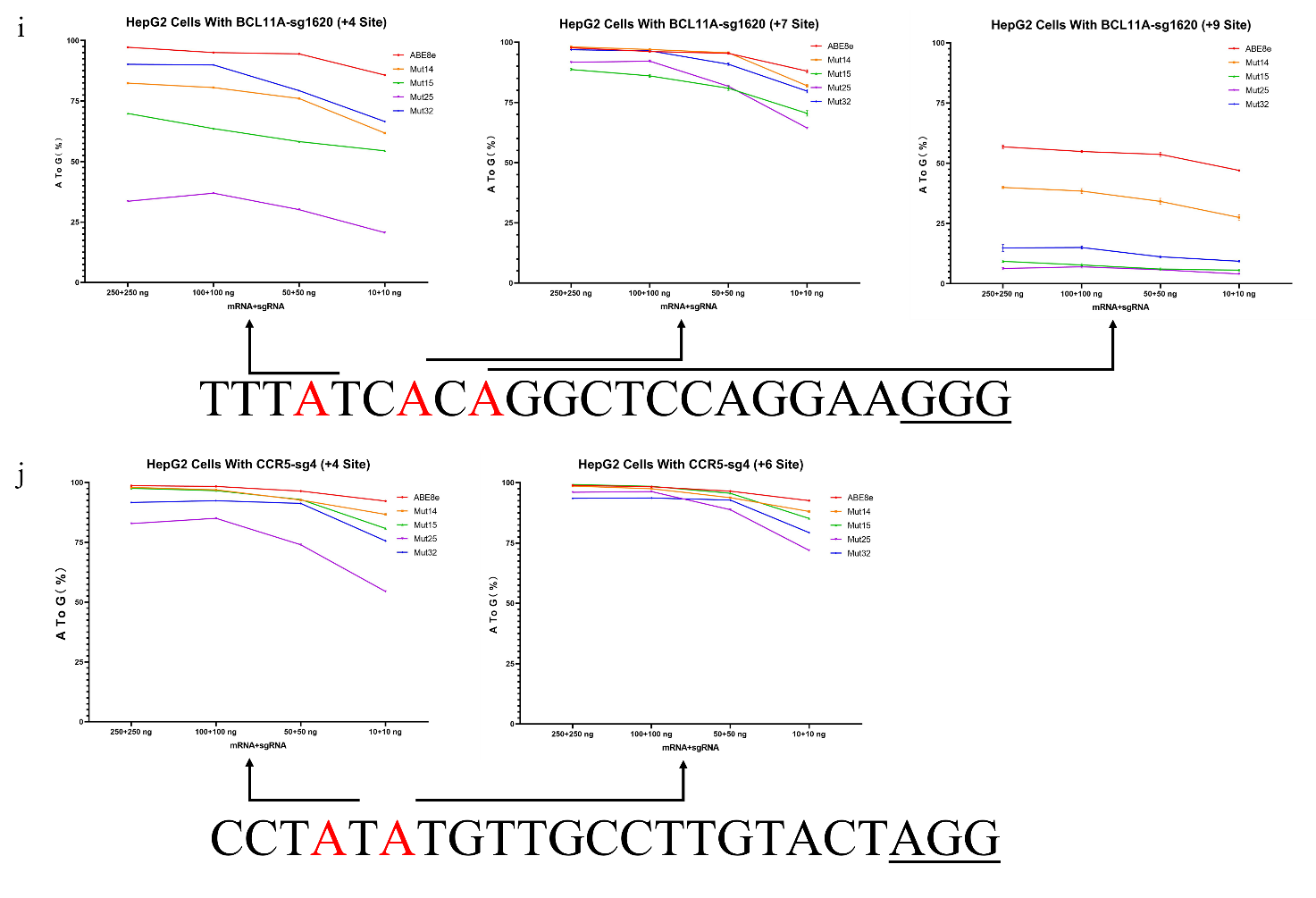


Figure S7 | **Efficiency data in detail of ABE variants by mRNA delivery with LNP.** Base editing efficiency of different mRNA doses at a. PCSK9-sg1 site in HEK293T and b. HepG2; c. CCR5-sg3 site in HEK293T and d. HepG2; e. CCR5-sg2 site in HEK293T and f. HepG2. g. AAVS1-sg2 site in HEK293T and h. HepG2; i. BCL11A-sg1620 site in HepG2; j. CCR5-sg4 site in HepG2. All data have three independent biological replicates (mean ± SD).


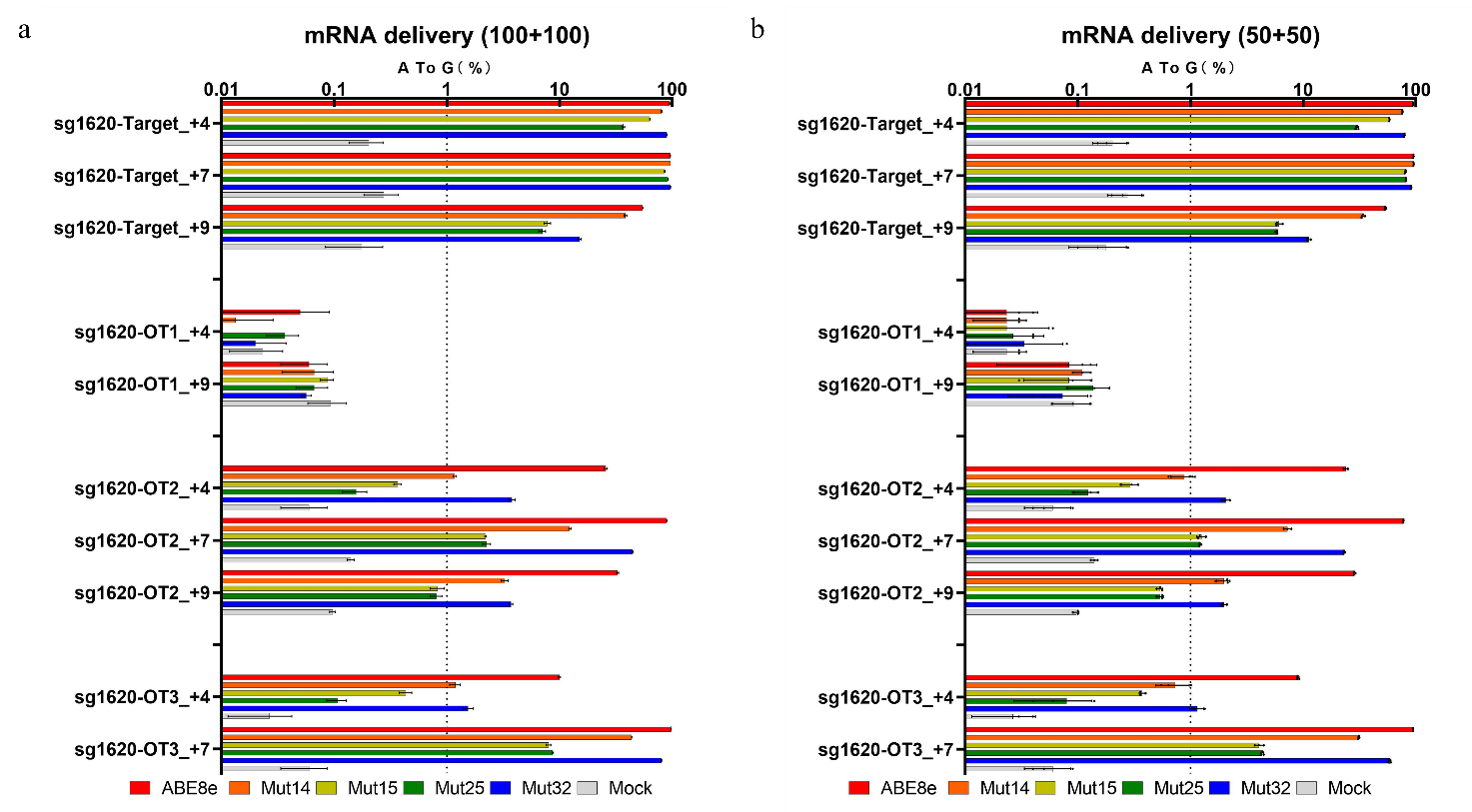


Figure S8 | **ABE variants’ mRNA delivery off-target data**. At the BCL11A-sg1620 site in HepG2 cells with a. 100 ng and b. 50 ng dosage. All data have three independent biological replicates (mean ± SD).


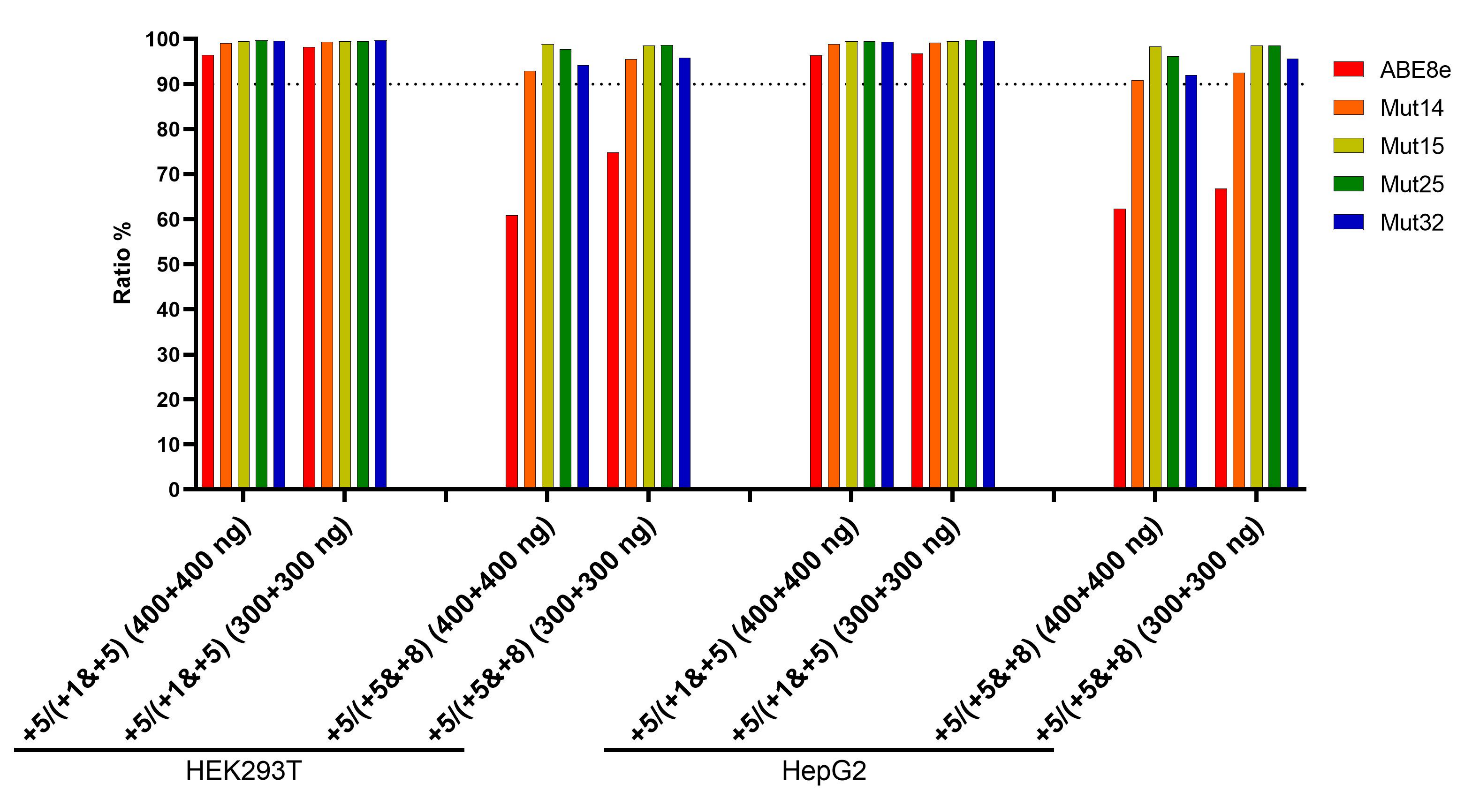


Figure S9 | **Editing purity analysis of therapeutic correction mediated by ABE variants.** The proportion of +5 on-target HEF-C282Y correction with mRNA by LNP delivery into HEK293T and HepG2. All the data have three independent biological replicates (mean ± SD).


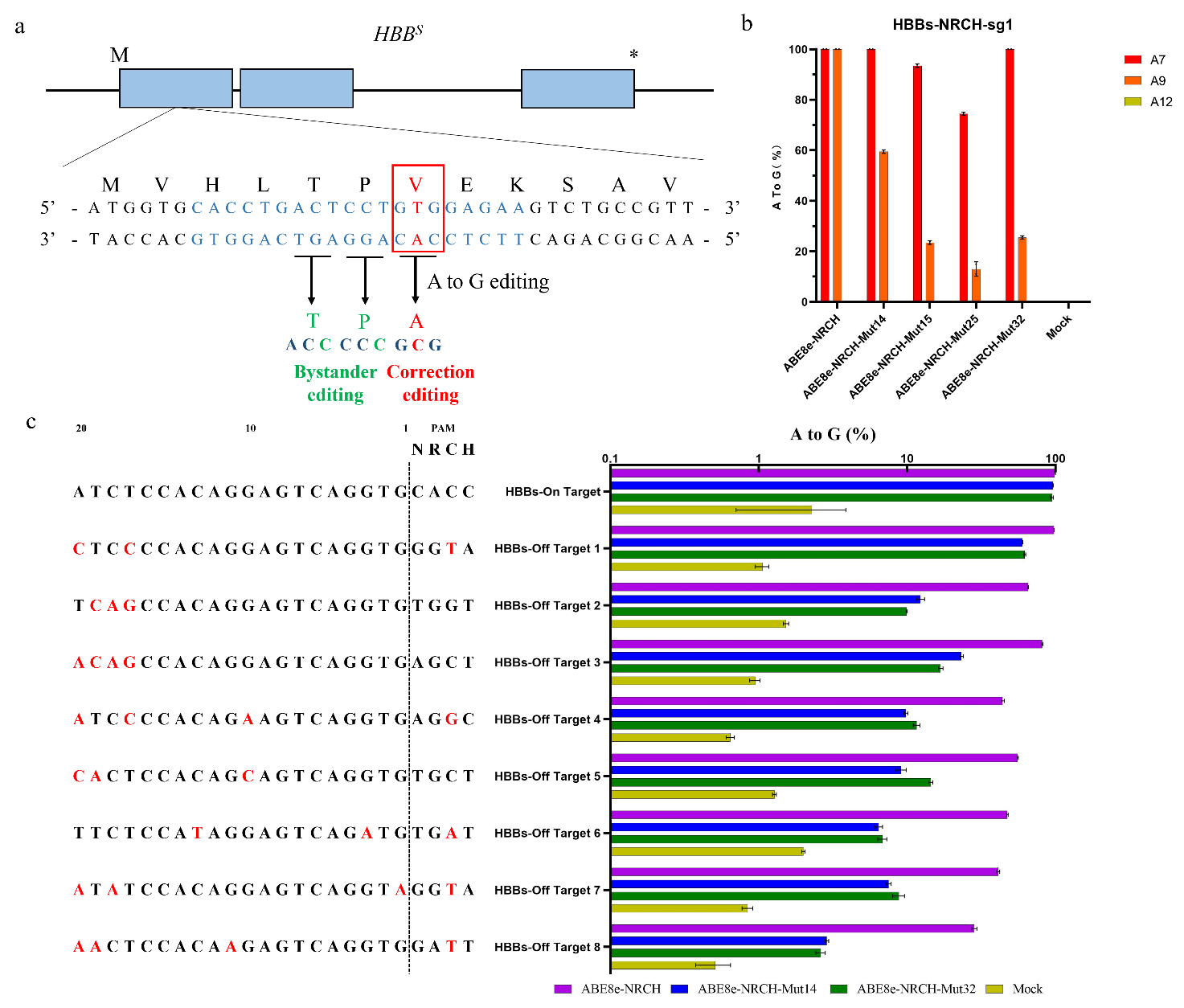


Figure S10 | Correction of HBB^S^ by ABE variant mRNAs. a. The mutation site of HBB^S^ in human genome and on-target correction editing as well as possible bystander editing. b. Correction outcome in HepG2 HBB^S^ disease model. c. The sequences of on-target and off-target sites are shown. The bar graphs show the total A-to-G reads within the editing windows. All the data have three independent biological replicates (mean ± SD).


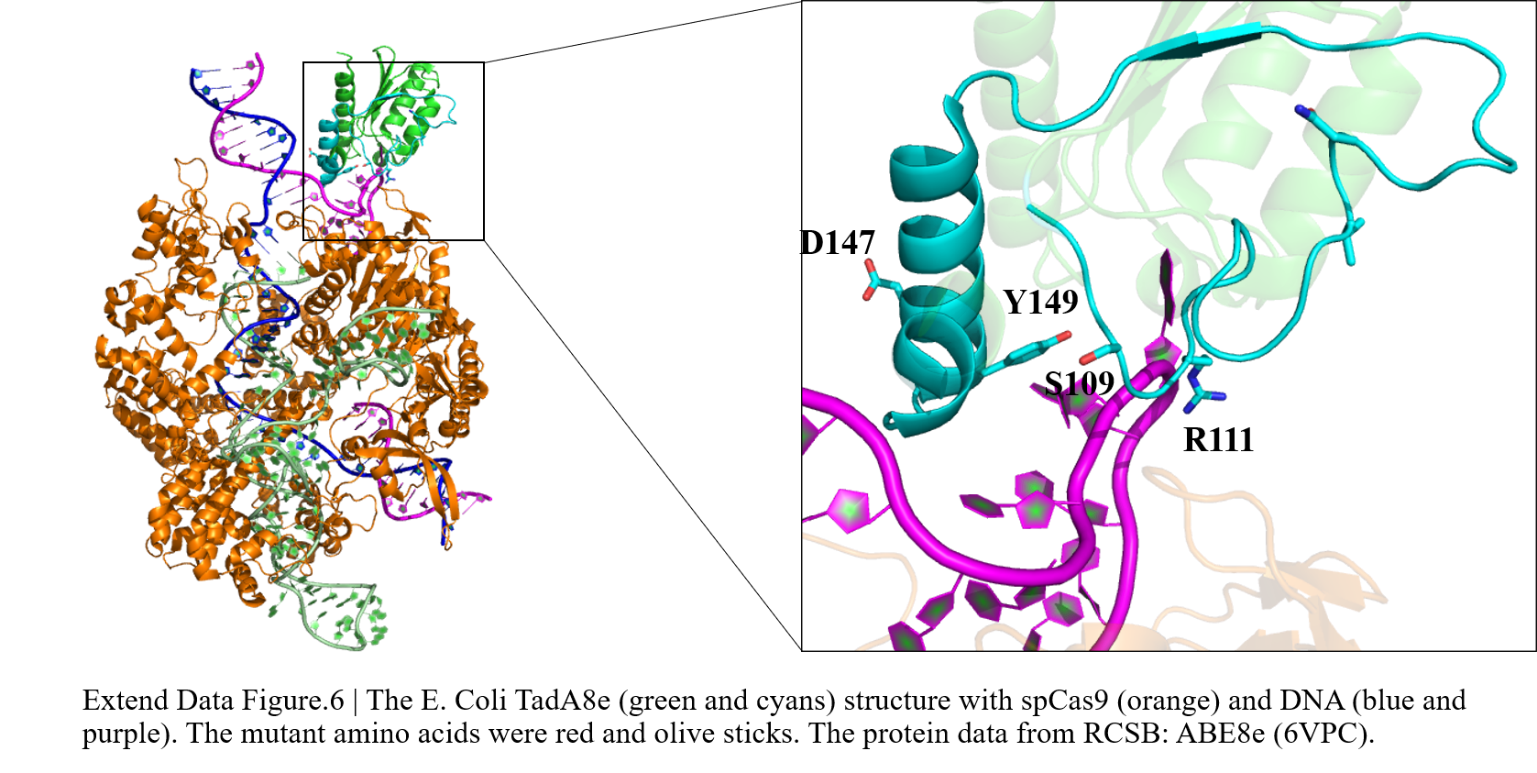


Figure S11 | The E. Coli TadA8e (green and cyan) structure with spCas9 (orange), sgRNA (palegreen), and DNA (blue and magenta). The mutant amino acids were shown as sticks. Elements are colored as follows: carbon, cyan; oxygen, red; nitrogen, dark blue. The structural data (PDB accession code: 6VPC).
